# Unmet health care needs among pregnant women during COVID‐19 pandemic and its determinants in Kurdistan province, Iran: A cross‐sectional study

**DOI:** 10.1002/hsr2.804

**Published:** 2022-09-01

**Authors:** Abdorrahim Afkhamzadeh, Azad Shokri, Hossein Safari, Elham Nouri, Amjad Mohamadi Bolbanabad, Shina Amirihosseini, Bakhtiar Piroozi

**Affiliations:** ^1^ Social Determinants of Health Research Center, Research Institute for Health Development Kurdistan University of Medical Sciences Sanandaj Iran; ^2^ Health Promotion Research Center Iran University of Medical Science Tehran Iran; ^3^ School of Nursing and Midwifery Qazvin University of Medical Sciences Qazvin Iran; ^4^ Student Research Committee Kurdistan University of Medical Sciences Sanandaj Iran

**Keywords:** access, COVID‐19, equity, unmet healthcare needs, utilization

## Abstract

**Background and Aims:**

The aim of this study was to assess unmet needs for health care and its determinants during COVID‐19 pandemic among pregnant women in Iran.

**Methods:**

This cross‐sectional study was performed among pregnant women in Kurdistan province in 2020 with a sample size of 800 people who were selected by multistage sampling method. Data were collected using a structured questionnaire that was completed through interviews. Also, multiple logistic regression was used to determine the relationship between independent variables and outcome variable. Statistical tests were performed using Stata software package.

**Results:**

The highest unmet needs for health care were related to dental services with 66%, rehabilitation services with 58.6%, and psychological services with 43.2% and the lowest were related to hospital services with 12%, midwifery services with 15.6%, and physician visit services with 39.1%. The most important reasons for unmet needs for health care were fear of getting COVID‐19 and the cost of the services. The variables of age group and spouse education for physician visit services; age group for midwifery services; age group, education and employment status for dental services; age group, supplementary insurance and economic status for rehabilitation services; and age group and economic status for psychological services were significantly associated with unmet needs for health care (*p* < 0.05).

**Conclusion:**

A significant percentage of health care needs of pregnant women was unmet, for which the fear of getting COVID‐19 and financial barriers were the main reasons.

## 1 INTRODUCTION

In any society, health is considered as a prerequisite for sustainable development in which women have an irreplaceable role as the main axis of public health.^1^ Pregnant women are one of the most vulnerable groups in any society. Receiving essential health care by this group is of special importance because they play a key role in the health of mother and child.^2^ It is widely accepted that the use of health care during pregnancy will reduce morbidity and mortality of mothers and infants. In other words, access to and use of health care services by pregnant mothers are of the most important factors in improving pregnancy outcomes.^3^, ^4^, ^5^ Understanding health needs, as well as unmet health needs and barriers to their use of health services, is an important key in developing strategies to improve the health of pregnant women.

One of the goals of health systems is to provide equal access to health care for people with equal needs. One way to measure equality of access to services is through reports of unmet needs for health care.^6^ Unmet healthcare needs may lead to poorer health for people who have dropped out from healthcare services. Unmet needs are a significant driver in creating health inequalities and reflect health needs for which services are not provided.^7^ Identifying the gap between “perceived health care needs” and “benefiting from health care” and finding the causes of this gap is very important to design targeted interventions to reduce this gap and improve access to health care services.^8^ Access to health services is influenced by various factors such as gender, age, marital status, knowledge, attitude, education, health beliefs, financial status, insurance status, health services cost, quality of provided care, distance to health care provider, general health status, the severity of the disease and disability.^9^, ^10^, ^11^ Crises, both natural and unnatural, can affect the pattern of health services use, the behavior of people and health care providers.^12^, ^13^, ^14^ The crisis caused by COVID‐19 pandemic is a global crisis and has led to the infection and death of millions of people in the world. In Iran, according to statistics, 7,237,156 people have been infected so far and 141,386 people have died as a result of this disease.^15^ COVID‐19 pandemic has affected the use of health services and has caused the shift of human resources and equipment of health sector towards the prevention, diagnosis, and treatment of this disease.^16^

A survey by WHO found that the highest disruptions in providing health care services during COVID‐19 were among low‐income countries, and it was estimated that reducing basic maternal and child health interventions could result in more than one million child deaths.^17^, ^18^ People may not receive health services for fear of getting COVID‐19 if they go to a health center or because of not having access to services due to lockdown and cancellation of or delay in providing nonemergency services.^16^

Stress, social distance, and limitations due to the current situation can affect the pattern of need and benefit of pregnant women from health services as part of society. Identifying the needs and factors affecting the use of health services and unmet health care needs is very important to formulate and implement targeted policies and interventions. Based on the literature review, no study has ever been done on assessing unmet health needs and its causes among pregnant women, as a vulnerable part of society, during the COVID‐19 crisis. In this regard, the present study was conducted to assess unmet health needs and their causes during the COVID‐19 pandemic among pregnant women in Iran (Kurdistan Province).

## 2 MATERIALS AND METHODS

### 2.1 Study participants and sampling

This was a cross‐sectional study. The study population included pregnant women in Kurdistan province in 2020, who were pregnant for at least 6 months. In the first stage, the sample size was determined 600 people using the following formula and taking into account *p* = 50% (benefiting from health services), *d* = 0.04 (accuracy rate) and *α* = 0.05 (type I error).

$$n=\frac{Z_{1-\frac{\alpha }{2}\times P(1-P)}^{2}}{d^{2}}=600.$$

Since the clustering method was used for sampling; therefore, to increase the sampling accuracy, the design effect was set at 1.33 and the final sample size was 800 people. Multistage sampling was used for choosing the samples. First, Sanandaj and Mariwan counties were randomly selected among 10 counties of the province, and then within each county, the county center and its villages were selected. In the next stage, 10 centers (five urban and five rural centers) were randomly selected by cluster random sampling from the comprehensive urban and rural health centers of each selected county. Then, among the pregnant women covered by each center, 40 pregnant women were randomly selected and after obtaining the addresses, a questionnaire was completed for them by a trained person by going to their door and through doing an interview.

### 2.2 Data collection

The required information was collected through the Utilization of Health Services Questionnaire (UHS), which is a valid and reliable questionnaire developed by Iranian National Institute of Health Research.^6^ The questionnaire consists of three parts: the first part covers demographic and background information including age, gender, occupation, education, employment status, type of basic insurance, supplementary insurance status and place of residence. The second part includes the economic status of the household and the third part focuses on whether the person (pregnant mother) needed health services or not, then the type of need is determined and in the next stage, the question is asked on whether she received the required services or not. Finally, the reasons for not receiving the required services (the *service was too expensive for us, the quality of service was low, there is no required medical specialty in this area, the waiting time was too long, it was far from my home, I did not have time, fear of getting COVID‐19 disease*) are clarified. The third section includes “Yes/No” questions. In this study, the need for health services was based on self‐reporting of the pregnant mother (illness and perceived need). Unmet needs were needs that the person realized existed but was unable to meet. It should be noted that the reminder period for outpatient health services including visits of doctors, midwives, dentists, psychologists, and rehabilitators was considered the last 4 weeks and for inpatient services including hospitalization was the last 6 months.

### 2.3 Statistical analysis

In this study, the asset index was applied to calculate the economic status. Using the principal composition analysis (PCA) method, first the variables that had the greatest impact on the variance of total variables were identified and then a new variable was created (SES) based on these variables. Using the mean of this variable, five quintiles were constructed that divided the population into five groups: very poor, poor, average, rich, and very rich. Multivariate logistic model, adjusted odds ratio, and confidence interval were used to determine the relationship between independent variables and the outcome variables (unmet health care needs). In the adjusted model, the variables that had *p* < 0.2 in the crude model were included in the model and other variables were removed from the model. All analyzes were performed in Stata 12.0 (Stata Corporation) and *p* < 0.05 was considered a statistical significance level.

### 2.4 Ethical considerations

The ethics committee of Kurdistan University of Medical Sciences approved the protocol of the study (No. IR.MUK.REC.1399.246). The researchers explained the research goals and protocol to the participants before their inclusion in the study, and written informed consent was obtained from all eligible participants who were willing to participate in the study.

## 3 RESULTS

A total of 716 out of 800 pregnant women in Kurdistan province participated in this study, of which 98% (703) were covered by basic health insurance and 8.5% (61) were covered by supplementary insurance. The majority of participants were in the 20–40 age group (74.8%), had high school education (44.1%), had a spouse with high school education (39.9%), were housewives (90.9%), and lived in the village (541.3%). In this study, 61.2% of mothers (438 individuals) needed health services and 94% of these mothers had at least one unmet need. The highest and lowest perceived needs were related to physician visit services with 35.3% (352 people) and psychological services with 6% (43 people), respectively.

**Figure 1:**
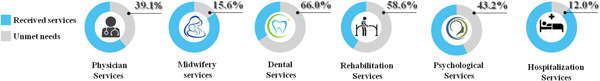
Unmet health care needs among pregnant women in Kurdistan province.

Also, the highest ratio of received services was related to hospital services with 87.9% (73 people) and the lowest ratio was for dental services with 34.0% (48 people). In contrast, the highest and lowest proportion of unmet needs were related to dental services with 66% (93 people) and hospital services with 12% (10 people) (Table 1 and Figure 1). In addition, “fear of getting COVID‐19 disease” and “cost of services” were reported as reasons for not using health services despite the need. For example, for dental services, 63% of people reported the cost of services and for inpatient services, 80% of people reported fear of getting COVID‐19 as a barrier to using the required health services. It should be noted that in the present study, none of the pregnant mothers had given up receiving their required services due to lack of physical access, lack of time, low quality of services or lack of relevant specialist (Table 1).

**Table 1:** Demographic characteristics, perceived needs, received services, and unmet needs by type of service in the population of pregnant women in Kurdistan province

	Physician visit				Midwifery services		Dental services		
	*N* (%)	Perceived need, *n* (%)	Received services	Unmet need	Perceived need, *n* (%)	Received services	Unmet need	Perceived need, *n* (%)	Received services	Unmet need
Age										
<20	20 (2.8)	4 (20.0)	1 (25.0)	3 (75.0)	6 (30.0)	6 (100.0)	0 (0.0)	1 (5.0)	0 (0.0)	1 (100.0)
20–30	231 (32.3)	72 (31.2)	51 (70.8)	21 (29.2)	4 (1.7)	3 (75.0)	1 (25.0)	44 (19.0)	11 (25.0)	33 (75.0)
30–40	304 (42.5)	101 (33.2)	60 (59.4)	41 (40.6)	33 (10.9)	24 (72.7)	9 (27.3)	74 (24.3)	24 (32.4)	50 (67.6)
>40	161 (22.5)	76 (47.2)	42 (55.3)	34 (44.7)	21 (13.0)	21 (100.0)	0 (0.0)	22 (13.7)	13 (59.1)	9 (40.9)
Insurance coverage										
Yes	703 (98.2)	243 (34.6)	154 (63.4)	89 (36.6)	63 (9.0)	54 (84.4)	9 (14.1)	140 (19.9)	48 (34.0)	92 (65.2)
No	13 (1.8)	10 (76.9)	0 (0.0)	10 (100.0)	1 (7.7)	0 (0.0)	1 (100.0)	1 (7.7)	0 (0.0)	1 (100.0)
Supplementary insurance coverage										
Yes	61 (8.5)	29 (47.5)	23 (79.3)	6 (20.7)	10 (16.4)	9 (90.0)	1 (10.0)	22 (36.1)	18 (81.8)	4 (18.2)
No	655 (91.5)	224 (34.2)	131 (58.5)	93 (41.5)	54 (8.2)	45 (83.3)	9 (16.7)	119 (18.2)	30 (25.5)	89 (74.8)
Education status										
Illiterate	21 (2.9)	14 (66.7)	10 (71.4)	4 (28.6)	1 (4.8)	1 (100.0)	0 (0.0)	12 (57.1)	4 (33.3)	8 (66.7)
Primary school	149 (20.8)	61 (40.9)	34 (55.7)	27 (44.3)	11 (7.4)	5 (45.5)	6 (54.5)	29 (19.5)	8 (27.6)	21 (72.4)
Middle school	160 (22.3)	59 (36.9)	32 (54.2)	27 (45.8)	24 (15.0)	23 (95.8)	1 (4.2)	32 (20.0)	5 (15.6)	27 (84.4)
High school	316 (44.1)	98 (31.0)	70 (71.4)	28 (28.6)	25 (7.9)	22 (88.0)	3 (12.0)	62 (19.6)	28 (45.2)	34 (54.8)
Diploma	70 (9.8)	21 (30.0)	8 (38.1)	13 (61.9)	3 (4.3)	3 (100.0)	0 (0.0)	6 (8.6)	3 (50.0)	3 (50.0)
Education status of the spouse										
Illiterate	17 (2.4)	3 (1.2)	0 (0.0)	3 (100.0)	1 (5.9)	0 (0.0)	1 (100.0)	0 (0.0)	…	…
Primary school	142 (19.8)	70 (49.3)	41 (58.6)	29 (41.1)	15 (10.6)	14 (93.3)	1 (6.7)	35 (24.6)	13 (37.1)	22 (62.9)
Middle school	178 (24.9)	43 (24.2)	14 (32.6)	29 (67.4)	26 (14.6)	21 (80.8)	5 (19.2)	16 (9.0)	4 (25.0)	12 (75.0)
High school	282 (39.4)	104 (36.9)	82 (78.8)	22 (21.2)	8 (2.8)	8 (100.0)	0 (0.0)	65 (23.0)	12 (18.5)	53 (81.5)
University degree	97 (13.5)	33 (34.0)	17 (51.5)	16 (48.5)	14 (14.4)	11 (78.6)	3 (21.4)	25 (25.8)	19 (76.0)	6 (24.0)
Job										
Has job	65 (9.1)	28 (43.1)	23 (82.1)	5 (17.9)	12 (18.5)	11 (91.7)	1 (8.3)	20 (30.8)	18 (90.0)	2 (10.0)
Householder	651 (90.9)	225 (34.6)	131 (58.2)	94 (41.8)	52 (8.0)	43 (82.7)	9 (17.3)	121 (18.6)	30 (24.8)	91 (75.2)
Place of residence										
Urban	349 (48.7)	107 (30.7)	58 (54.2)	49 (45.8)	38 (10.9)	33 (86.8)	5 (13.2)	64 (18.3)	20 (31.3)	44 (68.8)
Rural	367 (51.3)	146 (39.8)	96 (65.8)	50 (34.2)	26 (7.1)	21 (80.8)	5 (19.2)	77 (21.0)	28 (36.4)	49 (63.9)
Socioeconomic status										
Q1 (the poorest)	154 (21.5)	73 (48.7)	45 (61.6)	28 (38.4)	20 (13.3)	17 (85.0)	3 (15.0)	43 (27.7)	14 (32.6))	29 (67.4)
Q2	138 (19.3)	49 (35.5)	30 (61.2)	19 (38.8)	12 (8.7)	10 (83.3)	2 (16.7)	28 (20.3)	7 (25.0)	21 (75.0)
Q3	142 (19.8)	46 (32.4)	24 (52.2)	22 (47.8)	13 (9.2)	10 (76.9)	3 (23.1)	26 (18.3)	5 (19.2)	21 (80.8)
Q4	133 (18.6)	42 (31.6)	24 (57.1)	18 (42.9)	10 (7.5	9 (90.0)	1 (10.0)	24 (18.0)	9 (37.5)	15 (62.5)
Q5 (the richest)	149 (20.8)	43 (281.)	31 (72.1)	12 (27.9)	9 (5.9)	8 (88.9)	1 (11.1)	20 (13.7)	13 (65.0)	7 (35.0)
Reasons for unmet health care need										
Cost of service	…	…	…	55 (55.5)	…	…	1 (10.0)	…	…	82 (88.2)
Fear of getting COVID‐19	…	…	…	71 (71.7)	…	…	9 (90.0)	…	…	36 (38.7)
Waiting time	…	…	…	22 (22.2)	…	…	0 (0.0)	…	…	0 (0.0)
Distance	…	…	…	0 (0.0)	…	…	0 (0.0)	…	…	0 (0.0)
Total	**716**	**253 (35.3)**	**154 (60.9)**	**99 (39.1)**	**64 (8.9)**	**54 (84.4)**	**10 (15.6)**	**141 (19.7)**	**48 (34.0)**	**93 (66.0)**

	Rehabilitation services			Psychological services			Hospitalization services		
	Perceived need, *n* (%)	Received services	Unmet need	Perceived need, *n* (%)	Received services	Unmet need	Perceived need, *n* (%)	Received services	Unmet need
Age									
<20	1 (5.0)	0 (0.0)	1 (100.0)	1 (5.0)	0 (0.0)	1 (100.0)	0 (0.0)	…	…
20–30	26 (11.3)	9 (34.6)	17 (56.4)	25 (10.8)	10 (40.0)	15 (60.0)	22 (9.5)	18 (81.8)	4 (18.2)
30–40	23 (7.6)	6 (26.1)	17 (73.9)	8 (2.6)	7 (87.5)	1 (12.5)	31 (10.2)	28 (90.3)	3 (9.7)
>40	20 (12.4)	14 (70.0)	6 (30.0)	9 (5.6)	7 (77.8)	2 (22.2)	30 (18.6)	27 (90.0)	3 (10.0)
Insurance coverage									
Yes	68 (9.7)	29 (42.6)	39 (57.3)	42 (6.0)	24 (57.1)	18 (42.9)	82 (11.7)	73 (88.0)	9 (10.8)
No	2 (15.4)	0 (0.0)	2 (100.0)	1 (7.7)	0 (0.0)	1 (100.0)	1 (7.7)	0 (0.0)	1 (100.0)
Supplementary insurance coverage									
Yes	10 (16.4)	8 (80.0)	2 (20.0)	6 (9.8)	5 (83.3)	1 (16.7)	9 (14.5)	8 (88.9)	1 (11.1)
No	60 (9.2)	21 (35.0)	39 (65.0)	37 (5.6)	19 (51.4)	18 (48.6)	74 (11.3)	65 (87.8)	9 (12.2)
Education status									
Illiterate	3 (14.3)	0 (0.0)	3 (100.0)	1 (4.8)	0 (0.0)	1 (100.0)	2 (9.5)	0 (0.0)	2 (100.0)
Primary school	14 (9.4)	2 (14.3)	12 (85.7)	13 (8.7)	11 (84.6)	2 (15.4)	29 (19.5)	27 (92.1)	2 (6.9)
Middle school	5 (3.1)	4 (80.0)	1 (20.0)	15 (9.4)	4 (26.7)	11 (73.3)	26 (16.3)	24 (92.3)	2 (7.7)
High school	41 (13.0)	20 (48.8)	21 (51.2)	13 (4.1)	9 (69.2)	4 (30.8)	14 (4.4)	11 (78.6)	3 (21.4)
Diploma	7 (10.0)	3 (42.9)	4 (57.1)	1 (1.4)	0 (0.0)	1 (100.0)	12 (17.1)	11 (91.7)	1 (8.3)
Education status of the spouse									
Illiterate	3 (17.6)	0 (0.0)	3 (100.0)	0 (0.00)	…	…	0 (0.0)	…	…
Primary school	36 (25.3)	14 (38.9)	22 (61.1)	3 (2.1)	2 (66.7)	1 (33.3)	31 (21.8)	29 (93.5)	2 (6.5)
Middle school	3 (1.7)	3 (100.0)	0 (0.0)	16 (9.0)	9 (56.3)	7 (43.7)	24 (13.5)	22 (91.7)	2 (8.3)
High school	23 (8.2)	8 (34.8)	15 (65.2)	23 (8.2)	13 (56.5)	10 (43.5)	18 (6.3)	12 (66.7)	6 (33.3)
University degree	5 (5.2)	4 (80.0)	1 (20.0)	1 (1.0)	0 (0.0)	1 (100)	10 (10.3)	10 (100.0)	0 (0.0)
Job									
Has job	6 (9.2)	5 (83.3)	1 (16.7)	7 (10.8)	6 (85.7)	1 (14.3)	8 (12.1)	7 (87.5)	1 (12.5)
Householder	64 (9.8)	24 (37.5)	40 (62.5)	36 (5.5)	18 (50.0)	18 (50)	75 (11.5))	66 (88.0)	9 (12)
Place of residence									
Urban	38 (10.9)	21 (55.3)	17 (44.7)	19 (5.4)	9 (47.4)	10 (52.6)	46 (13.2)	42 (91.3)	4 (8.7)
Rural	32 (8.7)	8 (25.0)	24 (75.0)	24 (6.5)	15 (62.5)	9 (37.5)	37 (10.1)	31 (83.8)	6 (16.2)
Socioeconomic status									
Q1 (the poorest)	27 (17.6)	9 (33.3)	18 (66.7)	13 (8.7)	5 (38.5)	8 (61.5)	25 (16.7)	19 (76.0)	6 (24.0)
Q2	15 (10.9)	4 (26.7)	11 (73.3)	10 (7.2)	5 (50.0)	5 (50.0)	16 (11.6)	14 (87.5)	2 (12.5)
Q3	12 (8.5)	4 (33.3)	8 (66.7)	8 (5.6)	3 (37.5)	5 (62.5)	15 (10.5)	14 (93.3)	1 (6.7)
Q4	10 (7.5)	6 (60.0)	4 (40.0)	6 (4.5)	5 (83.3)	1 (16.7)	14 (10.5)	14 (100.0)	0 (0.0)
Q5 (the richest)	6 (4.0)	6 (100.0)	0 (100.0)	6 (3.9)	6 (100.0)	0 (0.0)	13 (8.6)	12 (92.3)	1 (7.7)
Reasons for unmet health care need									
Cost of service	…	…	23 (56.1)	…	…	12 (63.2)	…	…	5 (50.0)
Fear of getting COVID‐19	…	…	18 (43.9)	…	…	7 (36.8)	…	…	8 (80.0)
Waiting time	…	…	0 (0.0)	…	…	0 (0.0)	…	…	0 (0.0)
Distance	…	…	0 (0.0)	…	…	0 (0.0)	…	…	0 (0.0)
Total	**70 (9.8)**	**29 (41.4)**	**41 (58.6)**	**43 (6.0)**	**24 (55.8)**	**19 (43.2)**	**83 (11.6)**	**73 (87.9)**	**10 (12.0)**

Table 2 shows the determinants of unmet needs in the crude and adjusted model. In the crude model, the majority of variables were among the significant determinants of unmet needs. For instance, for physician visit services, women in the age group >35 years (odds ratio [OR] = 2.9, *p* < 0.001), without supplementary insurance coverage (OR = 2.7, *p* = 0.036) and housewives (OR = 3.3. *p* = 0.019) had higher chance of having unmet needs. However, those women whose spouses had higher education (OR = 0.65, *p* < 0.001) had lower odds of having unmet needs.

**Table 2:** Results of multivariate logistic regression for determinants of unmet health care needs in pregnant women in Kurdistan province by type of service

	Physician visit				Midwifery services		Dental services			
	OR (95% CI)^a^	*p* Value	OR (95% CI)^a^	*p* Value	OR (95% CI)^a^	*P* Value	OR (95% CI)^a^	*p* Value	OR (95% CI)^a^	*p* Value
Age										
**35**>	1.00		1.00		1.00		1.00		1.00	
**35**<	2.91 (1.72–4.91)	***<0.001***	2.10 (1.18–3.75)	***0.012***	0.18 (0.04–0.79)	***0.023***	0.28 (0.12–0.61)	***0.001***	0.13 (0.044–0.40)	***<0.001***
Supplementary insurance coverage										
Yes	1.00		1.00		1.00		1.00		1.00	
No	2.72 (1.07–6.94)	***0.036***	1.11 (0.29–4.21)	0.883	1.80 (0.20–16.03)	0.598	13.35 (4.19–42.57)	***<0.001***	4.15 (0.52–33.27)	0.18
Education status										
Middle school>	1.00		1.00		1.00		1.00		1.00	
Middle school<	0.69 (0.41–1.15)	0.152	0.99 (0.55–1.80)	0.152	0.50 (0.12–2.13)	0.346	0.36 (0.18–0.75)	***0.006***	0.34 (0.12–0.94)	***0.038***
Education status of the spouse										
Middle school>	1.00		1.00		1.00		1.00			
Middle school<	0.35 (0.21–0.58)	***<0.001***	0.49 (0.26–0.92)	***0.026***	0.79 (0.18–3.41)	0.752	0.95 (0.46–1.97)	0.894		
Job										
Has job	1.00		1.00		1.00		1.00		1.00	
Householder	3.30 (1.21–8.99)	***0.019***	2.69 (0.68–10.64)	0.157	2.30 (0.26–20.16)	0.451	27.30 (5.98–124.58)	***<0.001***	11.43 (1.31–99.85)	***0.028***
Place of residence										
Urban	1.00		1.00		1.00		1.00			
Rural	0.62 (0.37–1.03)	0.064	0.71 (0.41–1.21)	0.207	1.57 (0.41–6.01)	0.513	0.80 (0.39–1.61)	0.524		
Socioeconomic status										
Q3 (the richest)	1.00				1.00		1.00		1.00	
Q2	1.15 (0.65–2.04)	0.636			1.57 (0.27–9.05)	0.611	2.38 (1.09–5.20)	***0.029***	0.38 (0.12–1.26)	0.113
Q1 (the poorest)	1.68 (0.81–3.49)	0.163			2.55 (0.36–17.96)	0.347	4.20 (1.34–13.14)	***0.014***	1.58 (0.33–7.53)	0.567

	Rehabilitation services				Psychological services		Hospitalization services			
	OR (95% CI)^a^	*p* Value	OR (95% CI)^a^	*p* Value	OR (95% CI)^a^	*p* Value	OR (95% CI)^a^	*p* Value	OR (95% CI)^a^	*p* Value
Age										
**35**>	1.00		1.00		1.00		1.00		1.00	
**35**<	0.26 (0.09–0.75)	***0.013***	0.10 (0.01–0.62)	***0.012***	0.17 (0.03–0.88)	***0.035***	0.09 (0.01–0.59)	***0.013***	2.41 (0.63–9.30)	0.202
Supplementary insurance coverage										
Yes	1.00		1.00		1.00		1.00		1.00	
No	7.43 (1.44–38.21)	***0.016***	3.64 (1.68–7.91)	***0.022***	4.74 (0.50–44.57)	0.174	1.62 (0.07–38.10)	0.765	1.11 (0.12–9.92)	0.927
Education status										
Middle school>	1.00		1.00		1.00				1.00	
Middle school<	0.41 (0.14–1.22)	0.108	4.33 (0.81–23.22)	0.088	0.60 (0.16–2.21)	0.439			1.55 (0.40–6.02)	0.531
Education status of the spouse										
Middle school>	1.00				1.00				1.00	
Middle school<	0.91 (0.34–2.39)	0.843			1.16 (0.35–3.92)	0.807			3.48 (0.89–13.55)	0.073
Job										
Has job	1.00		1.00		1.00		1.00		1.00	
Householder	8.33 (0.92–75.65)	0.060	0.83 (0.03–25.03)	0.915	6.00 (0.66–54.99)	0.113	10.14 (0.50–66.52)	0.132	0.96 (0.11–8.68)	0.967
Place of residence										
Urban	1.00		1.00		1.00				1.00	
Rural	3.71 (1.33–10.32)	0.06	1.80 (0.39–5.61)	0.524	4.74 (0.50–44.57)	0.323			2.03 (0.53–7.82)	0.302
Socioeconomic status										
Q3 (the richest)	1.00				1.00		1.00			
Q2	6.70 (1.81–24.73)	***0.004***	4.76 (0.85–26.63)	***0.046***	14.3 (1.57–19.95)	***0.018***	10.14 (0.96–110.66)	***0.050***	6.30 (0.74–53.65)	0.092
Q1 (the poorest)	6.00 (1.15–31.23)	***0.033***	2.32 (0.28–19.12)	0.434	18.33 (1.51–32.87)	***0.022***	26.95 (1.47–49.39)	***0.026***	1.86 (0.11–32.01)	0.670

^a^ Crude model.

^b^ Adjusted model by removing all variables with *p* > 0.2.

In terms of dental services, women in higher age groups (OR = 0.28, *p* < 0.001) and with higher education (OR = 0.36, *p* = 0.006) had lower odds of unmet needs. However, the odds of having unmet needs were higher in groups without supplementary insurance coverage (OR = 13.3), in housewives (OR = 27.3) and in families with lower economic status (OR = 4.2) (*p* < 0.05). Similarly, the odds of unmet rehabilitation services were lower in older age groups (OR = 0.26) and were higher in women without supplementary insurance coverage (OR = 7.4), and in families with lower economic status (OR = 6.0) (*p* < 0.05). Also, for psychological services, the odds of unmet needs decreased with increasing age, having supplementary insurance coverage, and higher economic status (*p* < 0.05).

However, in the adjusted model after removing variables with *p* > 0.2, the variables of age group and spouse education for physician visit services; age group for midwifery services; age group, education and employment status for dental services; age group, supplementary insurance and economic status for rehabilitation services; and age group and economic status for psychological services were still significantly associated with unmet needs (*p* < 0.05).

## 4 DISCUSSION

Unmet health care needs are a simple tool for monitoring access to healthcare services and the extent of inequalities in access to health services. Unmet health needs are the gap between the services needed and the services actually received.^19^ The aim of this study was to assess the unmet health needs and their causes during the COVID‐19 pandemic among pregnant women in Iran (Kurdistan province).

According to the findings of this study, the most unmet health needs were related to dental services with 66%, rehabilitation services with 58.6%, and psychological services with 43.2%, and the lowest were related to hospital services with 12%, midwifery services with 15.6% and physician visit services with 39.1%. The most important reasons for not receiving the required services were “fear of getting COVID‐19” and “service cost.” Findings showed that a significant percentage of the need for health services, especially the need for dental and rehabilitation services, was not met and there was a deep gap between the perceived need and use of health services in the population of pregnant women. Although according to studies conducted in Iran, unmet health needs are significant, the findings of this study reported more unmet health needs than other studies in Iran.^6^, ^20^, ^21^ The reasons for such differences are due to the differences in the study population (pregnant mothers) along with the crisis and conditions caused by COVID‐19. According to previous studies, economic factors have been one of the serious barriers on the way of access to health services. As indicated by the findings of this study, the fear of getting COVID‐19 made people not to look for receiving health services.^20^, ^21^, ^22^, ^23^

Although basic health insurance organizations cover a huge percentage of the population in Iran, and according to statistics, more than 90% of the country's population has insurance, the country's insurance system has many challenges such as the number and type of services included in the insurance services package and depth of covering services costs. These issues have created a weakness in the efficiency of the insurance system in preventing households from facing catastrophic healthcare expenditures.^24^ Also, the significant difference between the tariff rate of the private and public sectors is another problem for the country's health system. Basic insurance, if the patient refers to the private sector, does not cover this difference in the tariff rate and all costs must be paid by the patient. In Iran, dental, midwifery, rehabilitation, and psychological services are not covered by basic health insurance and the cost of these services must be paid by the patients out of pocket.^22^, ^24^, ^25^

In Iran, most hospital services are provided by the public sector and most outpatient services (such as physician, and dentist visits as well as rehabilitation, psychology, and laboratory services) are provided by the private sector.^25^ In addition, the queue for receiving specialist doctor services is usually long. The reasons for this can be the low proportion of physicians to the population per capita and the inefficiency of the referral system. Although in the first level of care in Iran, which is provided by the public sector, routine pregnancy care is given to mothers in eight visits (two visits in the first half and six visits in the second half of pregnancy), a few numbers of people may receive such services due to regular absence of physicians, inadequate information about the existence of these services especially in urban areas, and lack of proper trust in the quality of services. According to a study conducted by Rezapour et al. in the general population of Iran, the highest unmet needs were reported for dental services (39.8%) and physician visits (32.4%). Lack of financial means and economic inefficiency were the two main reasons for unmet health needs.^21^ Based on the study of Motlagh et al. in Iran, one of the most important reasons for not using health services was lack of financial resources.^20^ The results of a systematic review by Moynihan et al. on the impact of COVID‐19 pandemic on benefiting from health services in 20 countries showed a median reduction of 37% in the use of health services. This decrease was 42% for visit services, 28% for hospitalization services, 31% for diagnostic services, and 30% for medical services.^16^ According to a study in France and Romania, unmet health care needs during pregnancy were caused mainly due to financial issues.^26^ As reported by “European Union statistics on income and living conditions” survey in 2014, an average of 26.5% of adults in European countries needed health care in the last 12 months. The index ranged from less than 10% in Cyprus and Norway to more than 40% in Ireland, Lithuania, Estonia, and Portugal. The main reasons for unmet health care needs were services cost and waiting time, respectively. On average, unmet needs were 12.3% for dental care, 5.9% for physician visits, and 2.7% for mental health. The index ranged from less than 5% for dental services in the Czech Republic, Cyprus, Malta, the Netherlands, and Norway to more than 30% in Portugal, Ireland, and Estonia. Also, it ranged from 0.7% in the United Kingdom to 33.1% in Iceland for mental health services.^27^ In another study based on “European Union statistics on income and living conditions” survey in 2018, the average unmet needs for medical care index was 3.2% in the last 12 months among adults in EU countries. There was a big difference between countries in terms of this index. The highest rate was in Estonia (19%), followed by Lithuania and Greece, and the lowest was in Austria, Spain, Malta, Germany, the Netherlands, and Luxembourg (less than 1%). The main reasons for these unmet needs were service cost, waiting list, and distance, respectively. People in low‐income quintiles had more unmet health care needs. According to this study, unmet needs for dental care was 4% in the last 12 months and the main reason was the service cost in all countries.^27^ According to the commonwealth fund international health policy survey, unmet needs for medical care among adults ranged from 7% in the United Kingdom to 33% in the United States, and the value of this index was significantly higher in lower income groups. In almost all countries, the main reason for unmet needs was reported to be the service cost. In the preceding study, unmet needs for dental care were reported by a larger segment of the population because dental services for adults are not covered by public insurance in most countries. The value of this index varied from 11% in the Netherlands and the United Kingdom to 28% in Canada and 32% in the United States.^27^ In another study by Pappa et al. in Greece, 10% of health needs were not met and the main reason was the high cost of services.^28^ In the study of Barman et al. in India, financial status was one of the main determinants of unmet health needs. According to a 2014 New Zealand Health Survey, 29% of adults reported unmet health care needs.^29^

Based on the adjusted model in this study, the level of unmet needs for medical services was associated with variables of higher age group and lower level of education of the spouse; the level of unmet needs for midwifery services was associated with a lower age group; and the level of unmet needs for dental services was linked with variables of lower age group, lower education and nonemployment of pregnant women. Also, unmet needs for rehabilitation services was connected to lower age group, lack of supplementary insurance, and lower economic status, and the level of unmet needs for psychological services was directly and significantly related to variables of lower age group and lower economic status. According to a study by Rezapour et al. in Iran, poor households were less likely to receive the services they needed.^30^

As indicated by the study of Motlagh et al. in Iran, the most important factors affecting the use of health services were economic status, age, employment status, insurance coverage, level of education, and household size.^20^ According to the study of Rezaei et al. in Iran, the economic status of the household, age, and level of education of the head of the household were among the factors affecting the use of dental services.^22^ A study in Canada also found that households with better economic status had fewer unmet health needs.^31^ According to a study by Haddad et al. which was carried out using data from the Demographic Health Survey of seven countries of Bangladesh, Cambodia, Cameroon, Nepal, Peru, Senegal, and Uganda, a significant relationship was found between antenatal care use and household wealth, female education and place of residence.^32^ The results of a study in France and Romania showed that household income status affected the likelihood of pregnant women ignoring the services they needed; this probability decreased with increasing income levels. Having basic health insurance in Romania and supplementary insurance in France reduced the odds of unmet health care needs significantly. People without supplementary insurance were two times more likely to have unmet health care needs. In Romania, the odds of having unmet health care needs were higher for low‐educated women, and women under 25 and over 40 were also more likely to have unmet health care needs. According to a study in India, women's level of education and household economic status were influential factors in the use of prenatal health care.^26^

## 5 STRENGTHS OF THE STUDY

This is the first study in Iran to calculate the unmet health care needs among pregnant women during the COVID‐19 crisis.

## 6 LIMITATIONS OF THE STUDY

In this study, information about perceived need and using health services was collected based on self‐reporting of pregnant women and may be accompanied by a reminder error, although the research team tried to reduce this error by shortening the reminder period for outpatient services to 1 month and for inpatient services to 6 months.

## 7 CONCLUSION

There is a deep gap between perceived need and receiving health services in the population of pregnant women; much of the perceived need for health services has been ignored. The main reasons for UNHC were “fear of getting COVID‐19” and “services cost”. Our findings confirmed the idea that the existence of health care alone does not guarantee its use. In the crisis caused by COVID‐19, designing and implementation of appropriate interventions to reduce barriers on the way of responding to perceived health services of pregnant women, as a vulnerable group, should be put immediately on the agenda of health policy makers.

## AUTHOR CONTRIBUTIONS

**Abdorrahim Afkhamzadeh**: Conceptualization; formal analysis; supervision; writing—original draft. **Azad Shokri**: Formal analysis; methodology; writing—review and editing. **Hossein Safari**: Methodology; writing—original draft; writing—review and editing. **Elham Nouri**: Data curation; writing – review and editing. **Amjad Mohamadi Bolbanabad**: Formal analysis; methodology; writing—review and editing. **Shina Amirihosseini**: Data curation; writing—review and editing. **Bakhtiar Piroozi**: Conceptualization; formal analysis; writing—original draft; writing—review and editing.

## CONFLICT OF INTEREST

The authors declare no conflict of interest.

## TRANSPARENCY STATEMENT

The corresponding author, Bakhtiar Piroozi, affirms that this manuscript is an honest, accurate, and transparent study, the important aspects of the study have not been omitted, and all discrepancies in the study have been explained.

## Data Availability

The data that support the findings of this study are available from the corresponding author (Bakhtiar Piroozi) upon reasonable request.
